# The use of needle holders in CTF guided biopsies as a dose reduction tool

**DOI:** 10.1002/acm2.12234

**Published:** 2017-11-29

**Authors:** Sandra Sarmento, Joana S. Pereira, Maria José Sousa, Luís T. Cunha, Anabela G. Dias, Miguel F. Pereira, Augusto D. Oliveira, João V. Cardoso, Luís M. Santos, João A.M. Santos, João G. Alves

**Affiliations:** ^1^ Medical Physics, Radiobiology and Radiation Protection Group IPO Porto Research Center (CI‐IPOP) Medical Physics Service Portuguese Oncology Institute of Porto (IPO Porto) Porto Portugal; ^2^ Universidade de Lisboa (UL) Instituto Superior Técnico (IST) Laboratório de Protecção e Segurança Radiológica (LPSR) Bobadela LRS Portugal; ^3^ UL‐IST Centro de Ciências e Tecnologias Nucleares (C^2^TN) Bobadela LRS Portugal; ^4^ Interventional Radiology Service Portuguese Oncology Institute of Porto (IPO Porto) Porto Portugal; ^5^ Instituto de Ciências Biomédicas Abel Salazar da Universidade do Porto Porto Portugal

**Keywords:** CTF‐guided biopsies, dosimeters, hand monitoring, interventional radiology, needle holders, radiation exposure

## Abstract

**Purpose:**

The purpose of this study was to evaluate the efficacy of needle holders in reducing staff hand exposure during biopsies guided by computed tomography fluoroscopy (CTF), through the analysis of data acquired during a detailed monitoring study, undertaken in parallel with an ongoing optimization process to reduce hand irradiation.

**Methods:**

Hand monitoring was performed with 11 extremity detectors, two per finger (base and tip) and one on the back of the wrist, for the left (dominant) hand, during two series of biopsies with comparable characteristics. The first series (47 biopsies) were performed with only quick‐check method (QC) and occasional side‐handle (SH) manipulation of the needle. The second series (63 biopsies) were performed after introducing needle holders (NH) in the course of an optimization process.

**Results:**

Choice of technique (QC, QC + NH, QC + SH) by the interventional radiologist (IR) was related to biopsy difficulty. Measured hand exposure was low (< 1 mSv) for all QC‐only procedures, and for most of the QC + NH procedures. Occasional side‐handle manipulation still occurred during challenging biopsies, so that 8% of biopsies in the second series accounted for ~70% of total fingertip dose (~90 mSv). The methodology used allowed a detailed insight into the dose reduction achievable with needle holders during real procedures, without the limitations of phantom measurements.

**Conclusions:**

Needle holders proved effective in reducing mean hand exposure during clinical procedures where real‐time manipulation was necessary. Occasional side‐handle manipulation was found to contribute disproportionately to hand exposure. This highlights the importance of individual hand monitoring during CTF guided procedures.

## 1 INTRODUCTION

Computed tomography (CT) is a useful imaging technique to guide interventional radiology procedures, allowing good visualization of small lesions and neighboring critical structures, as well as the planned needle path. The possibility of in room real‐time CT imaging (also known as CT fluoroscopy, CT fluoro or CTF) is an additional advantage, particularly useful in lung biopsies where respiratory motion causes lesion displacement.^1^, ^2^, ^3^ CTF guidance for lung biopsies provides high diagnostic accuracy with fewer complications.^3^ Some newer CT scanners offer the possibility of multislice CT guidance (MS‐CT guidance), which proved effective in reducing radiation doses to patient and staff.^4^ But CTF guidance is still essential when lesions are subject to major respiratory movements.^4^

The main concern with CTF is radiation exposure. The designation CTF is adopted in this work, because the name “CT fluoroscopy” can be misleading. As pointed out by Miller et al*,* CTF is not fluoroscopy at all: it is different from conventional fluoroscopy in both equipment and technique.^5^ In CTF guided biopsies, the needle advancement occurs in the imaging/irradiation plane, so direct manipulation of the needle during irradiation would put the hands in the direct beam, where the dose rate may be as high as 4 mGy/s.^6^ At this dose rate, the occupational limit of 500 mSv per year for the hands^7^, ^8^ would be exceeded in less than 3 min (or 6 procedures considering the mean CTF times in this study). The quick‐check (QC) method proposed by Silverman et al prevents direct hand irradiation by using intermittent imaging to check needle position, while needle advancement occurs during beam‐off.^9^ Alternatively, needle holders (NH) may be used to avoid direct manipulation of the needle during continuous viewing.^1^, ^2^, ^10^, ^11^ Dedicated needle holders have been developed^1^, ^10^, ^11^, ^12^ but many authors prefer metallic sponge forceps or towel clamps due to their widespread availability, lightweight, strength, ease of sterilization and relatively low cost.^2^, ^9^, ^13^, ^14^ If a towel clamp is used to grasp and manipulate the needle, the hand may be kept 10–20 cm away from the irradiation plane during needle advancement. CTF dose rates drop very rapidly with distance from the scan plane.^6^ Other protective devices can be used in combination with needle holders, such as radiation attenuation gloves, lead drapes placed on the patient, and angular beam modulation (ABM). ABM means the CT scanner automatically turns off the irradiation beam during a pre‐selected part of the tube rotation.^15^ Not all manufacturers offer an ABM option. Nevertheless, it is important to remember that CTF has the potential for very high hand exposures, and operators should be mindful of this when performing these procedures.

All these protective options have been tested on phantoms,^1^, ^12^, ^15^, ^16^, ^17^ but phantom studies are limited: hand movements cannot be reproduced, and it is not possible to take into account the complexity of the procedures (including the difficulties introduced by protective measures themselves). Intermittent imaging has obvious limitations when dealing with respiratory motion, while needle holders have been associated to decreased tactile feedback and grip, as well as bending of thinner biopsy needles.^2^, ^9^, ^13^, ^14^, ^18^, ^19^ Because of such limitations, it is likely that interventional radiologists (IRs) will resort occasionally to manual manipulation of the side handle (SH) of the co‐axial guiding needle (side‐handle manipulation), as described by Buls et al.^20^ This puts the IR's hand very close to the irradiation beam, and may lead to high exposures near the fingertips.^21^

It is difficult to perform measurements of hand exposure during actual CTF guided procedures. Moreover, to evaluate the effect of protective measures like using needle holders, it would be necessary to compare success rates for similar biopsies performed with different techniques. IR's prefer real‐time visualization for smaller lesions, particularly when respiratory motion is a concern (e.g., lung biopsies). Even in abdominal biopsies, preference for the quick‐check method is associated with larger sized lesions.^9^ So far, the most comprehensive study is that of Irie et al, which compared finger doses per procedure (measured with ring dosimeters) using needle holders of different lengths, with and without a protective lead plate, for 55 procedures (mostly in the chest area).^10^ In the studies of Carlson et al^14^ and Paulson et al*,* 22 most procedures were performed using the quick‐check method alone. Silvermann et al estimated hand exposure for abdominal biopsies performed with needle holders, assuming the hands were 10 cm away from the beam.^9^ Recently, Kim et al reported per procedure ring dosimeter readings with a mean value of 0.76 mSv, for CTF guided biopsies performed using 22 cm surgical forceps as needle holders.^3^ Buls et al performed a large survey (82 procedures), measuring exposure at the back of both hands, without needle holders: the quick‐check (QC) method was used most of the time, with occasional side‐handle (SH) manipulation when necessary.^20^ The median hand dose measured by Buls et al was 0.76 mSv per procedure (maximum 7.3 mSv).^20^ However, finger doses may be 20 times higher than at the back of the hand when side‐handle (SH) manipulation is used.^21^

Other recent studies have focused on patient doses,^4^, ^23^ or assessment of recently introduced scanner features, like MS‐CT^4^ and iterative reconstruction.^24^ Because the total length irradiated during CTF is very small, patient effective doses associated with CTF are lower than those of a diagnostic CT scan.^6^ CTF used for needle positioning typically accounts for ~15% of the total DLP (dose length product) of a CTF guided procedure.^23^, ^25^ Peri‐interventional acquisitions (such as the initial helical CT scan acquired to determine the optimal access, and the lesion examination after the intervention) account for ~85% of the total DLP in CTF guided biopsies.^23^ On the other hand, since irradiation involves multiple rotations at the same position, patient skin doses may be a concern with CTF.^6^

The purpose of this work was to assess and improve occupational exposure, particularly the hand exposure of the IR, which was monitored in detail during CTF guided biopsies, in parallel with an ongoing optimization process to reduce hand irradiation. The routine use of needle holders (NH) was introduced as part of this optimization process, and therefore hand exposure was measured for two series of biopsies, before (before NH) and after (after NH) the introduction of needle holders. Approximately 80% of all biopsies performed were chest biopsies (lung or mediastinum). The results obtained provide a useful insight into the dose reduction achievable with improvised needle holders (20 cm towel clamps).

## 2 MATERIALS AND METHODS

**Figure 1:**
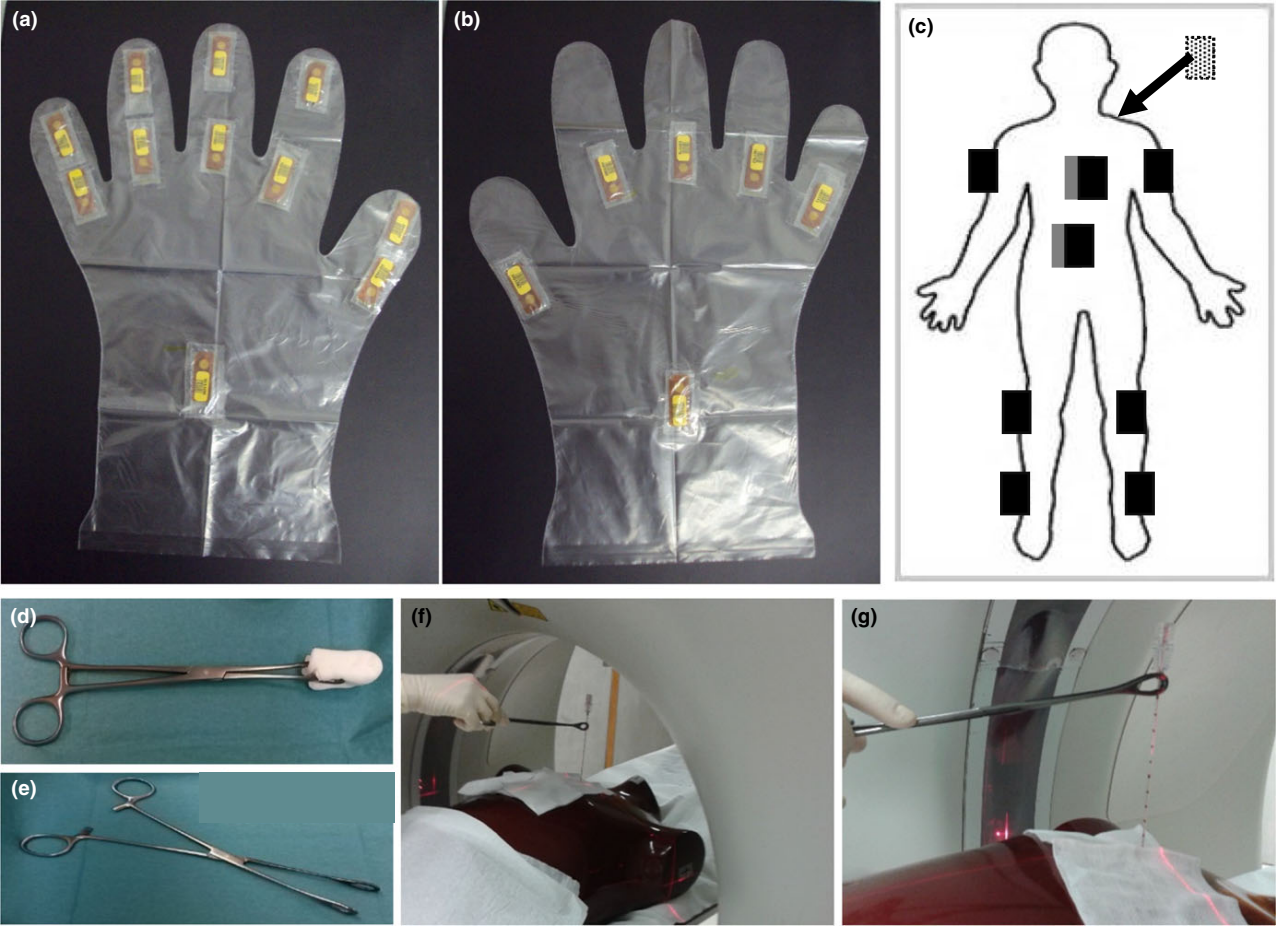
Detector locations and needle holders. Detector locations: on the left (a) and right (b) hands and body (c) of the IR (gray rectangles – WB dosimeters under the apron; dotted rectangle –WB dosimeter on the back). Photographs of the towel clamp used as an improvised needle holder (d, e). Use of the towel clamp as a needle holder simulated on a phantom (f,g).

Detailed hand monitoring was performed using the methodology described by Pereira et al.^26^ The location of the in room viewing monitor favors left‐handed needle manipulation, therefore the left hand is usually the most exposed. A thin plastic glove was prepared for the left hand, containing casings for the placement of 11 extremity detectors, two per finger (base and tip) and one on the back of the wrist, as shown in Fig. 1(a). Another glove was prepared for the right hand, with only six casings for detectors, one per finger (at the base) and the 6th at the back of the wrist, as shown in Fig. 1b). These gloves were tested before use, and found not to reduce hand mobility, dexterity or sensitivity in any way.^26^

A secondary objective of this detailed monitoring was to characterize the typical dose distributions of CTF guided procedures, to optimize individual monitoring. Whole body (WB) dosimeters were also placed at 12 different locations over and under the lead apron, as shown in Fig 1(c).

The WB dosimeters consist of the Harshaw 8814 card and holder containing two LiF:Mg,Ti (TLD‐100) detectors, with adequate filtration for the measurement of *H*_p_(10) and *H*_p_(0.07). The operational quantities *H*_p_(10) and *H*_p_(0.07) are recommended for assessment of effective dose and equivalent dose to local skin, respectively, defined as the dose equivalent to soft tissue at a depth of 10 mm and 0.07 mm below a specified point on the body.^7^, ^27^ The extremity detectors used in the gloves were of the Ext‐Rad type with LiF:Mg,Cu,P (TLD‐100H), for the measurement of *H*_p_(0.07) in routine individual monitoring.

All detectors were calibrated and read out by an approved dosimetry service (ADS, as defined in report RP160 of the European Commission^27^) which is also a provider of individual monitoring services. This allows direct comparison with ring dosimeter measurements from routine individual monitoring. Dosimeters were read using two Harshaw 6600 readers. The extremity dosimeters were calibrated in terms of *H*_p_(0.07) using a N120 X‐ray beam incident on an ISO rod phantom, and the WB dosimeters were calibrated in terms *H*_p_(10) and *H*_p_(0.07) using a ^137^Cs beam incident on a ISO water phantom.^28^, ^29^, ^30^, ^31^ The minimum limit of detection is 0.02 mSv in terms of *H*_p_(10) and *H*_p_(0.07) for WB detectors, and 0.07 mSv in terms of *H*_p_(0.07) for the extremity detectors.

All biopsies were performed by the same experienced interventional radiologist (IR), using a Toshiba Asteion four‐slice scanner and 120 V, 0.75 s rotation time and 8 mm beam collimation. 40 mA was used for most biopsies, and increased to 50 mA whenever necessary (usually for abdominal biopsies). The IR wears a wrap‐around lead apron (0.35 mm lead equivalent at the back, 0.70 mm at the front) and a thyroid shield (0.5 mm lead equivalent). As specified by the hospital's radiation safety program, both hands are normally monitored with ring dosimeters, and a whole body dosimeter is worn under the lead apron. These three dosimeters are read monthly, and are separate from this study.

The typical biopsy procedure is very similar to that described by Buls et al.^20^ The patient is brought into the CT room, the procedure is explained and a consent form signed. The patient is then positioned for a preliminary CT scan, restricted to the region where the lesion was previously detected. Once the lesion is located, a plane (slice position) is chosen and the couch moved to the appropriate position. The position indicated by the laser lights is marked with a radio‐opaque marker, and a single axial scan is acquired. The distance from the marker to the lesion is measured on the console, and a needle course is plotted. A sterilized drape is placed on the patient, the biopsy region is sterilized and local anaesthesia is administered.

Only the IR remains in the CT room during irradiation. The radiology technologist operates the CT scanner from the main console outside the CT room, with communication via the audio system.

The optimization process started with the acquisition parameters, to reduce the tube current to the lowest possible value. All the data presented here were obtained with optimized acquisition parameters. Initially, the IR wore attenuation gloves and, whenever the quick‐check (QC) method proved insufficient, the biopsy needle was grasped by the side handle (SH), as described by Buls et al.^20^ A total of 47 biopsies were performed with this methodology—these constitute the first series, named “before needle holder” (“before NH”).

In a second series, named “after needle holder” (“after NH”), an improvised needle holder (20 cm towel clamp, shown in Figs. 1(d) and 1(e)] was introduced, which should keep the hands at least 10 cm away from the radiation beam. In this second series with a total of 63 biopsies, attenuation gloves were no longer used, due to decreased tactile feedback with the needle holder. The quick‐check method (QC) was still preferred whenever possible, and constituted the first approach in all biopsies. Needle holders were used only after QC had been attempted (QC+NH), and only in the part of the biopsy where real‐time manipulation was particularly important. There were still situations where the IR chose to use side‐handle manipulation in combination with quick check (QC + SH), sometimes after using NH as well. The technique chosen was registered for each procedure of the second series, and CTF time for SH (T_SH) and NH (T_NH) use was estimated from visual observation of the procedure combined with recorded CTF times. Detailed monitoring was performed for all CTF guided biopsies of both series, regardless of the technique used, on randomly selected days.

## 3 RESULTS

Patient statistics and biopsy type were similar for both series of measurements, as summarized in Tables 1 and 2, respectively. Maximum *H*_p_(0.07) reading for the second series was 42.89 mSv for the left hand, and 0.87 mSv for the right hand. This confirms that the left hand is dominant for needle manipulation, independent of the location of the IR relative to the CT couch (Table 3).

**Table 1:** Patient statistics (age, weight, height, sex) for both series of biopsies, presented as mean (minimum – maximum)

	Age (y)	W (kg)	H (cm)	M/F (total number)
Before NH	65 (34–89)	67 (43–100)	166 (152–188)	23 M/24 F (47 total)
After NH	63 (17–84)	71 (45–109)	165 (148–195)	36 M/27 F (63 total)

**Table 2:** Biopsy statistics for both series

	Anatomical location of biopsy			CTF beam‐on time	Value of I chosen	
	Chest	Abdomen	Other‐bone	Mean (min–max)	50 mA	40 mA
Before NH	36 (76%)	6 (13%)	5 (11%)	26.0s (5.5s–85.2s)	7 (15%)	40 (85%)
After NH	51 (80%)	6 (10%)	6 (10%)	35.0s (6.6s–101.8s)	9 (14%)	54 (86%)

**Table 3:** Location of the IR relative to the CT couch, during the second series of biopsies

	Left	Right	Changed sides during biopsy
Number of biopsies	36	26	1

**Figure 2:**
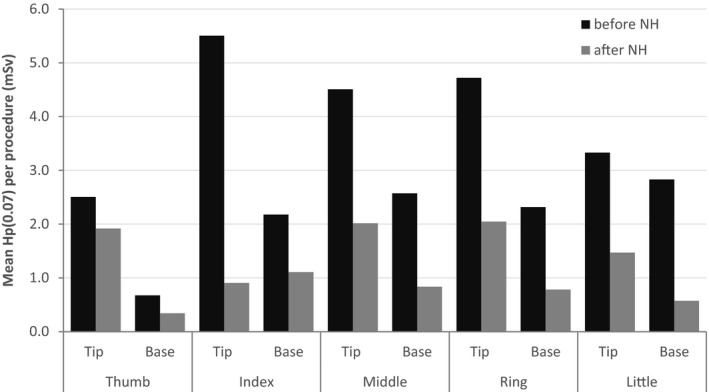
Mean values of H_p_(0.07) per procedure, measured on the left hand, at the tip and base of each finger, during the first series of biopsies (before NH) and during the second series of biopsies (after NH).

The mean *H*_p_(0.07) per procedure is compared for the left hand and both series of biopsies in Fig. 2. The introduction of needle holders resulted in a considerable reduction in left hand exposure. This decrease did not result from increased right hand manipulation, because the maximum right hand exposure also decreased from 3.89 mSv in the first series to 0.87 mSv in the second series. Right hand exposure was already low, with mean finger doses not exceeding 0.2 mSv per procedure in the first series. Therefore, right hand exposure will not be discussed further.

**Figure 3:**
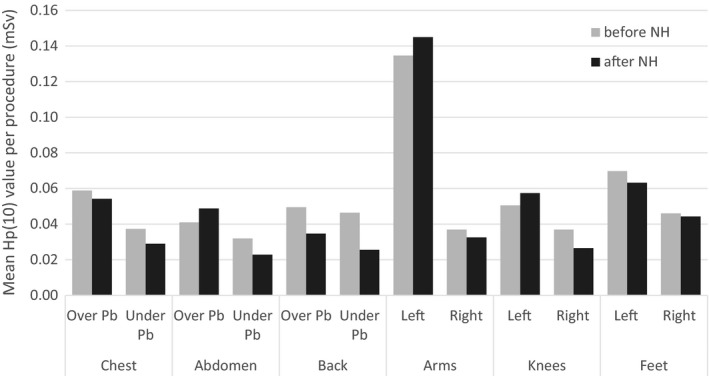
Mean values of H_p_(10) per procedure, measured during the first series of biopsies (before NH) and during the second series of biopsies (after NH). Acquisition parameters were similar for both series.

Mean *H*_p_(10) values on the chest, abdomen, back, arms, knees and feet are similar before and after the introduction of needle holders, as shown in Fig. 3.

**Figure 4:**
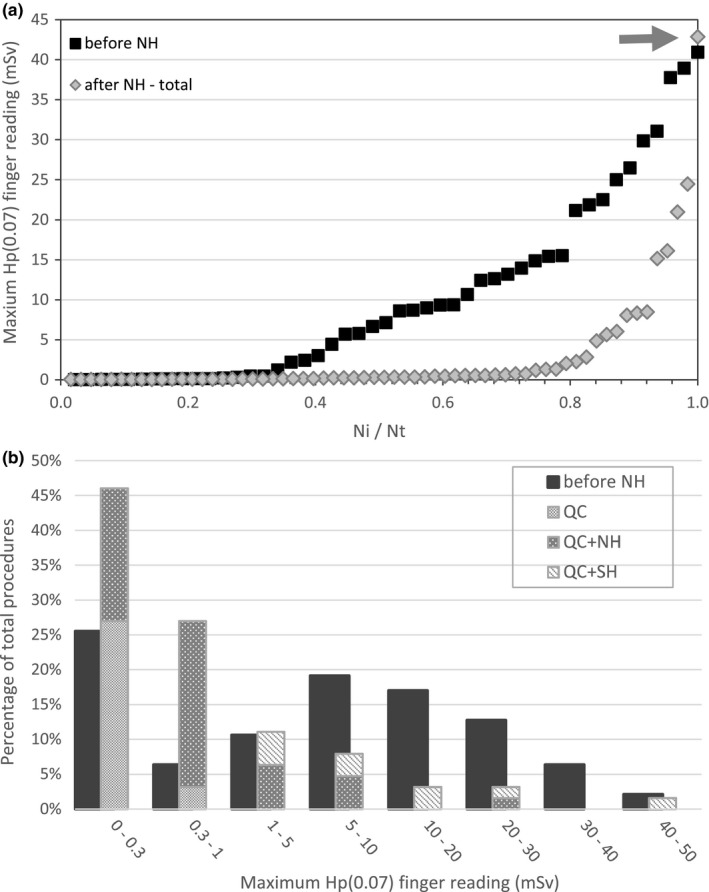
H_p_(0.07)max for both series of biopsies. (a) procedures of each series were numbered (Ni = 1,2,*…*) in the order of increasing value of H_p_(0.07)max, up until the total number of procedures in the series, Nt (Nt = 47 before NH; Nt = 63 after NH); H_p_(0.07)max values are presented as a function of Ni/Nt. (b) the same data is presented in a different manner, as percentage (%) of procedures (in each series) with H_p_(0.07)max in each interval; procedures of the second series (after NH) are divided by technique, with height of column representing % of each technique (%QC, %QC + NH, %QC + SH), and total height of column corresponding to added percentages (%QC + %QC + NH + %QC + SH), or total percentage of procedures with H_p_(0.07)max in each interval for the second series.

The maximum values of *H*_p_(0.07) in each procedure, *H*_p_(0.07)max, are compared in Figs. 4(a) and 4(b) for both series, and detailed for the second series in Table 4. Biopsies performed solely with the quick‐check technique (QC) are associated to low hand exposure [Table 4, Fig 4(b)]. The percentage of low exposure (0–1 mSv) biopsies in the first series is approximately the same as the percentage of QC biopsies in the second series. It seems reasonable to conclude that the percentage of biopsies performed with QC alone remained approximately the same.

**Table 4:** Hand doses per procedure, according to technique, for the second series of biopsies (after NH)

	# (%)	Anatomical location	Maximum *H*_p_(0.07) registered (mSv)	*H*_p_(0.07) range^a^ (mSv) [mean (min–max)]
QC	19 (30%)	16 chest	0.35	0.05 (0.01–0.19)
		1 abdomen		
		2 other‐bone		
QC + NH	35 (56%)	27 chest	24.47	1.52 (0.03–23.81)
		4 abdomen		
		4 other‐bone		
QC + SH	9 (14%)	8 chest	42.89	13.07 (1.73–42.22)
		1 abdomen		
Total	63 (100%)	51 chest	42.89	2.73 (0.01–42.22)
		6 abdomen		
		6 other‐bone		

a *H*_p_(0.07) range: difference between maximum and minimum value of *H*_p_(0.07) measured across the hand during each biopsy.

Interestingly, the highest value of *H*_p_(0.07)max is approximately the same for both series, as indicated by an arrow in Fig. 4. This is consistent with the fact that side‐handle manipulation (SH) was considered necessary in a small number of biopsies, even after the introduction of needle holders. SH is clearly associated with high hand exposure (Fig. 4, Table 4).

The effect of introducing needle holders is clearly seen in Fig. 4(a) and 4(b): intermediate exposures in the first series (5–15 mSv) are replaced by very low exposures in the second series (0–1 mSv). Moreover, *H*_p_(0.07)max seems to decrease even in the higher exposure group of biopsies (> 20 mSv). The combination of these two effects results in a lower mean exposure per procedure (Fig. 2).

Some biopsies performed with needle holders (QC + NH) resulted in *H*_p_(0.07)max values of 5–30 mSv, which suggests the needle holders were gripped nearer the needle, or rotated closer to the beam. This is reflected also in the variation in exposure across the hand (Table 4). The greatest range of variation is associated with side‐handle manipulation (SH), where hand exposure is strongly influenced by spatial location and finger positioning.

**Figure 5:**
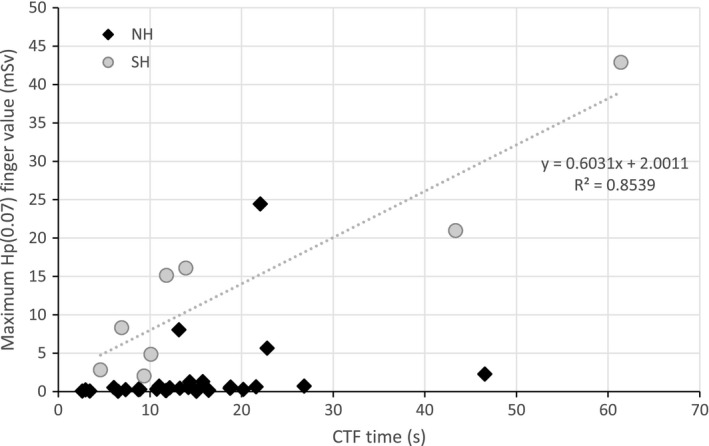
Maximum H_p_(0.07) values plotted as a function of the corresponding CTF time, for needle holder (NH) and side‐handle (SH) manipulation; the CTF times given are estimates for the duration of NH and SH manipulation (T_NH and T_SH), and the fit shown pertains to H_p_(0.07)max as a function of T_SH.

In Fig. 5, *H*_p_(0.07)max is plotted as a function of T_SH. The linear fit to this data suggests a dose rate nearly 10 times lower than expected for direct hand irradiation. SH manipulation seems to keep the hand outside the beam most of the time, just not far enough to avoid high hand exposures. *H*_p_(0.07)max for QC + NH procedures is shown in Fig. 5 for comparison: with QC + NH procedures, the higher values of *H*_p_(0.07)max are not related to increased manipulation times (T_NH). There is little correlation between *H*_p_(0.07)max and CTF times, except when SH occurs, as reflected by the R^2^ values obtained for linear fits (0.0673 for T_NH, 0.8539 for T_SH, 0.0378 for T_QC, and 0.3054 for T_CTF).

## 4 DISCUSSION

This study has some limitations, because hand exposure was assessed for only one IR, one CT scanner, a specific patient population, and certain types of CTF guided procedures. However, this also reduces the number of variables, allowing greater focus on the introduction of needle holders. Comparison of hand exposure with different techniques (QC, QC + NH or QC + SH) is difficult, because choice of technique is related to biopsy difficulty. The methodology used in this study was to compare two series of biopsies pertaining to the same patient population, with similar patient statistics (Table 1). Each series constitutes a typical sample of CTF guided biopsies in this hospital, with similar scanner and acquisition parameters (Table 2). The selection process was the same for both series: hand exposure was assessed for all biopsies on randomly chosen days. Therefore, in this scenario, it seems reasonable to assume that the distribution of biopsy difficulty was similar for both series, and that hand exposures can be compared.

As shown in Fig. 2, the introduction of needle holders reduced hand exposure considerably for this IR. The highest mean exposure in the second series is ~2 mSv per procedure, at the tips of the middle and ring finger (left hand). This IR performs approximately 200 CTF guided biopsies a year, so with the use of needle holders hand exposure is below the annual regulatory limit of 500 mSv.^7^, ^8^ With the mean hand exposure observed in the first series of biopsies, the same limit of 500 mSv would be reached after only 100 procedures.

The introduction of needle holders was associated with a 9 s increase in the mean value of CTF beam‐on time (Table 2). Assessment of patient doses is outside the scope of this paper, which focuses on the hand exposure of the IR and aims to prevent it from exceeding the regulatory limits. Nevertheless, for a complete analysis it is important to estimate the impact of this increase (in CTF time) on patient skin doses. Considering the dose rate estimated by Keat of 4 mGy/s in the direct beam,^6^ the maximum patient skin dose associated with CTF in this work was 408 mGy or 0.4 Gy (corresponding to the maximum beam on time of 102 s in Table 2). This is well below the threshold for deterministic effects (2 Gy for transient erythema^32^). A 9 s increase in CTF beam‐on time results in an estimated increase in patient skin dose of 36 mGy (~0.04 Gy), which is not a cause for concern. Moreover, it may be a transitory effect resulting from lack of familiarity with needle holders, which had just been introduced. The 9 s increase in CTF beam‐on time did not noticeably affect mean procedure time (which includes prepping the patient and collecting the biopsy sample).

Evaluation of biopsy success rates is equally outside the scope of this work, because the use of needle holders remained optional during the second series of biopsies. As shown in Table 4, the IR chose to use QC + SH during the more challenging biopsies in the second series (~14%). Therefore, these biopsies were performed with the same technique used in the first series. In this situation, biopsy success rates (e.g., accuracy of advancement and needle placement) are assumed equal for both series. If the IR had been constrained to use only QC or QC + NH in the second series, than the success rate for these 14% biopsies would have had to be assessed. Without this constraint, the percentage of QC + SH procedures in the second series of biopsies gives some indication of how frequently this IR felt the needle holders to be a limitation.

The data presented in Fig. 4 suggests that, during the second series of biopsies, needle holders replaced side‐handle manipulation in most procedures where real‐time manipulation was necessary. As expected, most QC + NH procedures involve low hand exposure, so the high number of procedures performed with needle holders instead of side‐handle manipulation resulted in the significant reduction in hand exposure seen in Fig. 2. As seen in Fig. 4, nearly half the procedures in the first series of biopsies resulted in *H*_p_(0.07)max > 5 mSv, compared to less than 20% of procedures in the second series. Some QC + NH biopsies were still associated with *H*_p_(0.07)max > 5 mSv, but this occurred mostly in the first month of the second series of measurements, and may have been associated with inexperience in the use of needle holders.

The percentage of biopsies performed with the quick‐check method (QC) alone appears to have remained the same, despite availability of needle holders (Fig. 4). This is good, because QC results in very low hand exposures, so its substitution by QC + NH would confer no advantage in terms of radiological protection. Use of QC alone is probably related to less challenging procedures. Real‐time visualization is more important when respiratory motion is a concern, but the need for real‐time manipulation is not determined by anatomical area alone (Table 4). Other factors are clearly involved, such as size and accessibility of lesion.

In the second series of biopsies, there were only five values of *H*_p_(0.07)max higher than 10 mSv, mostly associated with side‐handle manipulation. *H*_p_(0.07)max values cannot be added directly, because they occur at different locations for each biopsy. *H*_p_(0.07) values were added for each measuring location [see Fig. 1(a)], for the five biopsies with *H*_p_(0.07)max higher than 10 mSv. The most exposed locations were the tips of the thumb, middle and ring fingers of the left hand where the mean exposure was ~18 mSv/procedure (cumulative total ~ 90 mSv). These five biopsies with *H*_p_(0.07)max > 10 mSv correspond to about 8% of the biopsies in the second series, but were responsible for nearly 70% of the total fingertip dose in this series. More importantly, this occurrence of *H*_p_(0.07)max > 10 mSv remained approximately constant, at 6%–8% of biopsies (mostly associated with SH), until this study ended, 1 year after the introduction of needle holders into routine practice. Needle holders are associated with loss of tactile feedback and grip, which may be critical during particularly challenging biopsies, even for an experienced IR well acquainted with their use.

This data highlights the importance of constant individual monitoring of hand exposure. The need for side‐handle manipulation in ~7% of biopsies is easily overlooked in a busy routine, and left out of dose estimates based on approximate hand distance to the beam. Our results show that these rare occurrences contribute disproportionally to overall hand exposure, and therefore there is always a potential for dose escalation, either through inexperience or overconfidence. Moreover, individual attitudes toward risk vary between individuals, and concerns over patient safety also play a role. But methods of extremity monitoring during CTF guided biopsies need to be improved, because fingertip dose is not correctly assessed by ring dosimeters.^21^

The methodology used in this study, comparing hand exposure for two series of biopsies, is different from previous reports of staff dose studies, and allows a more detailed insight into the dose reduction achievable with needle holders in a clinical scenario. In this study, some biopsies could be performed with the quick‐check method alone, and for these there was no advantage in using needle holders. When real‐time manipulation was necessary, needle holders proved extremely effective at reducing overall hand exposure, by greatly reducing the number of procedures where SH was used. This reduced the mean hand doses per procedure to less than half the values observed for the first series of biopsies (Fig. 2).

Unfortunately, the methodology used in this study requires an ongoing optimization process, so opportunities for such comparisons are rare. Nevertheless, it would be interesting to have similar data for other types of needle holders, different IRs, different biopsy types, and CT scanners with angular beam modulation (ABM). Hopefully, the data presented here will lead to greater awareness of the potential for escalation of hand exposure, and prompt further studies during ongoing optimization processes.

## 5 CONCLUSIONS

Hand exposure was measured for two series of biopsies with comparable characteristics. This allows a detailed insight into the effect of protection measures during real procedures, without the limitations of phantom measurements.

Needle holders proved extremely effective at reducing hand irradiation during CTF guided biopsies when real‐time manipulation is necessary. Use of the quick‐check method alone leads to even lower exposures—therefore, the quick‐check method should be preferred if real‐time manipulation is not essential. In this study, the introduction of needle holders did not alter the percentage of procedures performed with the quick‐check method alone, nor did it completely prevent side‐handle manipulation of the needle. However, availability of needle holders greatly reduced the number of procedures where side‐handle manipulation was used, and this lowered the mean hand exposure considerably (Fig. 2).

When needle holders are available, occasional high hand exposure related to side‐handle manipulation during challenging biopsies is a rare occurrence (~8% of biopsies in the second series), but contributes disproportionately to hand exposure (nearly 70% of total hand dose in this study). This highlights the importance of constant individual hand monitoring, to avoid dose escalation through inexperience or overconfidence.

## CONFLICT OF INTEREST

The authors declare that they have no conflicts of interest.

## References

[1] Kato R , Katada K , Anno H , Suzuki S , Ida Y , Koga S . Radiation dosimetry at CT fluoroscopy: physician's hand dose and development of needle holders. Radiology. 1996;201:576–578. https://doi.org/10.1148/radiology.201.2.8888264. 8888264 10.1148/radiology.201.2.8888264

[2] Daly B , Templeton PA . Real‐time CT fluoroscopy: evolution of an interventional tool. Radiology. 1999;211:309–315. https://doi.org/10.1148/radiology.211.2.r99ma51309. 10228508 10.1148/radiology.211.2.r99ma51309

[3] Kim GR , Hur J , Lee SM , et al. CT fluoroscopy‐guided lung biopsy versus conventional CT‐guided lung biopsy: a prospective controlled study to assess radiation doses and diagnostic performance. Eur Radiol. 2011;21:232–239. 20730613 10.1007/s00330-010-1936-y

[4] Prosch H , Stadler A , Schilling M , et al. CT fluoroscopy‐guided vs. multislice CT biopsy mode‐guided lung biopsies: accuracy, complications and radiation dose. Eur J Radiol. 2012;81:1029–1033. 21752567 10.1016/j.ejrad.2011.01.064

[5] Miller DL , Vañó E , Bartal G , et al. Occupational radiation protection in interventional radiology: a joint guideline of the Cardiovascular and Interventional Radiology Society of Europe and the Society of Interventional Radiology. Cardiovasc Intervent Radiol. 2010;33:230–239. 20020300 10.1007/s00270-009-9756-7 PMC2841268

[6] Keat N . Real‐time CT and CT fluoroscopy. Br J Radiol. 2001;74:1088–1090. 11777764 10.1259/bjr.74.888.741088

[7] ICRP Publication 103: The 2007 Recommendations of the International Commission on Radiological Protection; 2007. Ann ICRP 37:1‐332 10.1016/j.icrp.2007.10.003 18082557

[8] COUNCIL DIRECTIVE 2013/59/EURATOM of 5 December 2013 laying down basic safety standards for protection against the dangers arising from exposure to ionising radiation, and repealing Directives 89/618/Euratom, 90/641/Euratom, 96/29/Euratom, 97/43/Euratom a. Off J Eur Union. 2014;57. https://doi.org/10.3000/19770677.l_2014.013.eng.

[9] Silverman SG , Tuncali K , Adams DF , Nawfel RD , Zou KH , Judy PF . CT fluoroscopy‐guided abdominal interventions: techniques, results, and radiation exposure. Radiology. 1999;212:673–681. 10478231 10.1148/radiology.212.3.r99se36673

[10] Irie T , Kajitani M , Itai Y . CT fluoroscopy‐guided intervention: marked reduction of scattered radiation dose to the physician's hand by use of a lead plate and an improved II device. J Vasc Interv Radiol. 2001;12:1417–1421. 11742017 10.1016/s1051-0443(07)61701-1

[11] Irie T , Kajitani M , Matsueda K , Arai Y . Biopsy of lung nodules with use of II device under intermittent CT fluoroscopic guidance: preliminary clinical study. J Vasc Interv Radiol. 2001;12:215–219. 11265886 10.1016/s1051-0443(07)61828-4

[12] Stoeckelhuber BM , Leibecke T , Schulz E , et al. Radiation dose to the radiologist's hand during continuous CT fluoroscopy‐guided interventions. Cardiovasc Intervent Radiol. 2005;28:589–594. 16132384 10.1007/s00270-005-0104-2

[13] Daly B , Templeton PA , Krebs TL , Carroll K , Wong‐You‐Cheong JJ . Evaluation of biopsy needles and prototypic needle guide devices for percutaneous biopsy with CT fluoroscopic guidance in simulated organ tissue. Radiology. 1998;209:850–855. 9844686 10.1148/radiology.209.3.9844686

[14] Carlson SK , Bender CE , Classic KL , et al. Benefits and safety of CT fluoroscopy in interventional radiologic procedures. Radiology. 2001;219:515–520. 11323481 10.1148/radiology.219.2.r01ma41515

[15] Hohl C , Suess C , Wildberger JE , et al. Dose reduction during CT fluoroscopy : phantom study of angular beam modulation. Radiology. 2008;246:519. 18227544 10.1148/radiol.2462061968

[16] Nawfel RD , Judy PF , Silverman SG , Hooton S , Tuncali K , Adams DF . Patient and personnel exposure during ct fluoroscopy – guided interventional procedures. Radiology. 2000;216:184. 10.1148/radiology.216.1.r00jl39180 10887246

[17] Neeman Z , Dromi SA , Sarin S , Wood BJ . CT fluoroscopy shielding : decreases in scattered radiation for the patient and operator. J Vasc Interv Radiol. 2006;17:1999–2004. 17185699 10.1097/01.RVI.0000244847.63204.5F PMC2408953

[18] Daly B , Wong‐you‐cheong JJ , Wang SS . Percutaneous abdominal and pelvic interventional procedures using CT fluoroscopy guidance. Am J Radiol. 1999;173:637–644. 10.2214/ajr.173.3.10470894 10470894

[19] de Mey J , Op de Beeck B , Meysman M , et al. Real time CT‐fluoroscopy: diagnostic and therapeutic applications. Eur J Radiol. 2000;34:32–40. 10802205 10.1016/s0720-048x(00)00157-1

[20] Buls N , Pagés J , de Mey J , Osteaux M . Evaluation of patient and staff doses during various CT fluoroscopy guided interventions. Health Phys. 2003;85:165–173. 12938963 10.1097/00004032-200308000-00005

[21] Sarmento S , Pereira J , Sousa MJ , et al. Gafchromic XR‐QA2 film as a complementary dosimeter for hand‐monitoring in CTF‐guided biopsies. J Appl Clin Med Phys. 2016;17:316–327. 26894341 10.1120/jacmp.v17i1.5725 PMC5690215

[22] Paulson EK , Sheafor DH , Enterline DS , McAdams HP , Yoshizumi TT . CT fluoroscopy–guided interventional procedures: techniques and radiation dose to radiologists. Radiology. 2001;220:161–167. 11425990 10.1148/radiology.220.1.r01jl29161

[23] Kloeckner R , Dos Santos DP , Schneider J , Kara L , Dueber C , Pitton MB . Radiation exposure in CT‐guided interventions. Eur J Radiol. 2013;82:2253–2257. 24050880 10.1016/j.ejrad.2013.08.035

[24] Grosser OS , Wybranski C , Kupitz D , et al. Improvement of image quality and dose management in CT fluoroscopy by iterative 3D image reconstruction. Eur Radiol. 2017;. https://doi.org/10.1007/s00330-017-4754-7. 10.1007/s00330-017-4754-7 28168371

[25] Teeuwisse WM , Geleijns J , Broerse JJ , Obermann WR , Van Persijn van Meerten EL . Patient and staff dose during CT guided biopsy, drainage and coagulation. Br J Radiol. 2001;74:720–726. 11511497 10.1259/bjr.74.884.740720

[26] Pereira MF , Alves JG , Sarmento S , et al. Preliminary assessment of the dose to the interventional radiologist in fluoro‐CT‐guided procedures. Radiat Prot Dosimetry. 2011;144:448–452. 21112883 10.1093/rpd/ncq418

[27] RADIATION PROTECTION NO 160 Technical Recommendations for Monitoring Individuals Occupationally Exposed to External Radiation. European Commission; Luxembourg; 2009. 10.1093/rpd/ncq295 20959338

[28] ISO 4037‐1:1996 ‐ X and Gamma Reference Radiation for Calibrating Dosemeters and Doserate Meters and for Determining Their Response as a Function of Photon Energy – Part 1: Radiation Characteristics and Production Methods. Geneva: International Organization for Standardization

[29] Alves JG , Calado AM , Cardoso JV , Santos LM . Energy and angular dependence of the personal dosemeter in use at ITN‐DPRSN. Radiat Meas. 2008;43:641–645.

[30] ISO 12794:2000 ‐ Nuclear Energy – Radiation Protection – Individual Thermoluminescence Dosemeters for Extremities and Eyes. Geneva: International Organization for Standardization

[31] ISO 4037‐3:1999 ‐ X and Gamma Reference Radiation for Calibrating Dosemeters and Doserate Meters and for Determining Their Response as a Function of Photon Energy – Part 3: Calibration of Area and Personal Dosemeters and the Measurement of Their Response. Geneva: International Organization for Standardization

[32] ICRP Publication 85: Avoidance of Radiation Injuries from Medical Interventional Procedures ; 2001. Ann. ICRP 30 (2) 10.1016/S0146-6453(01)00004-5 11459599

